# Orquestic regulation of neurotransmitters on reward-seeking behavior

**DOI:** 10.1186/1755-7682-7-29

**Published:** 2014-06-16

**Authors:** Oscar Arias-Carrión, Xanic Caraza-Santiago, Sergio Salgado-Licona, Mohamed Salama, Sergio Machado, Antonio Egidio Nardi, Manuel Menéndez-González, Eric Murillo-Rodríguez

**Affiliations:** 1 Unidad de Trastornos del Movimiento y Sueño (TMS), Hospital General Dr. Manuel Gea González, Mexico City, Mexico; 2 Unidad de Trastornos del Movimiento y Sueño (TMS), Hospital General Ajusco Medio, Mexico City, Mexico; 3 Toxicology Department and Medical Experimental Research Center (MERC), Faculty of Medicine, Mansoura University, Mansoura, Egypt; 4 Panic and Respiration, Institute of Psychiatry of Federal University of Rio de Janeiro, Rio de Janeiro, Brazil; 5 Physical Activity Neuroscience Physical Activity Sciences Postgraduate Program, Salgado de Oliveira University, Niterói, Brazil; 6 Neurology Unit, Hospital Álvarez-Buylla, Mieres, Spain; 7 Laboratorio de Neurociencias Moleculares e Integrativas, Escuela de Medicina, División Ciencias de la Salud, Universidad Anáhuac Mayab, Mérida, Yucatán, Mexico

**Keywords:** Dopamine, Orexin, Serotonin, Galanin, Histamine, Endocannabinoids, Reward-seeking behavior, Drug addiction

## Abstract

The ventral tegmental area is strongly associated with the reward system. Dopamine is released in areas such as the nucleus accumbens and prefrontal cortex as a result of rewarding experiences such as food, sex, and neutral stimuli that become associated with them. Electrical stimulation of the ventral tegmental area or its output pathways can itself serve as a potent reward. Different drugs that increase dopamine levels are intrinsically rewarding. Although the dopaminergic system represent the cornerstone of the reward system, other neurotransmitters such as endogenous opioids, glutamate, γ-Aminobutyric acid, acetylcholine, serotonin, adenosine, endocannabinoids, orexins, galanin and histamine all affect this mesolimbic dopaminergic system. Consequently, genetic variations of neurotransmission are thought influence reward processing that in turn may affect distinctive social behavior and susceptibility to addiction. Here, we discuss current evidence on the orquestic regulation of different neurotranmitters on reward-seeking behavior and its potential effect on drug addiction.

## Introduction

Rewards are defined operationally as those objects which we will work to acquire through allocation of time, energy, or effort; that is, any object or goal that we seek [1]. Generally, rewards are conditionally learned based upon their positive influence on survival or reproduction. Food and water serve vegetative needs and are therefore generally considered primary rewards. Money, which allows us access to food and improves our chance for reproduction, is a more abstract reward.

In this review, a stimulus is defined to be a reward insofar as it positively reinforces actions. That is, if upon retrieving an object an animal is more likely to repeat those behaviors that lead to the object in the future, then the object is designated to be positive reinforcing and hence a reward. Because rewards are defined so broadly, it is apparent that they may span a wide range of modalities. Still, organisms cannot pursue all possible rewards at any given moment in time. Different possibilities must be valued and chosen through direct comparison [2]. Due to this requirement, it has been proposed that there exists a single neural system which processes rewards of all modalities and thereby functions as a common scale through which diverse rewards may be contrasted [3]. However, here we discuss current evidence for the orquestic regulation of the different neurotransmitters on reward-seeking behavior and its potential effect on drug addiction.

### Drugs and natural reward

One issue that needs verification is whether drugs and natural rewards activate the same populations of neurons. Although there is overlap in the brain regions affected by natural rewards and drugs of abuse [4], similar overlap in neural populations that are affected by natural rewards and drugs can not be confirmed yet [5,6]. Based on previous data, can we understand drug addiction through studying natural reward? Recent evidence suggests that exposure to some non-drug rewards can impart “protection” from drug rewards. For example, sugar and saccharin can reduce self-administration of cocaine and heroin [7].

Several studies revealed that drug abuse usually begins by increasing the interest of individuals in natural rewards (sensitisation). Later on this interest decreases with prolonged drug consumption (compulsion). This paradox remains unexplained by current theories of addiction. The incentive sensitisation theory is viewed as a promising approach to this paradox, although it provides no mechanism to explain the decrease in interest of natural rewards as time exposure to a drug increases. Recently, Anselme described a model called the anticipatory dynamics model (ADM) that suggests a pivotal role of anticipation and attention in motivational interactions [8]. In addition to relying on strong neuropsychopharmacological data, the ADM provides an original conception of motivational specificity. This theory can be recognized as an extension of the incentive-sensitisation theory which hypothesizes how drugs interact with natural rewards.

**Figure 1:**
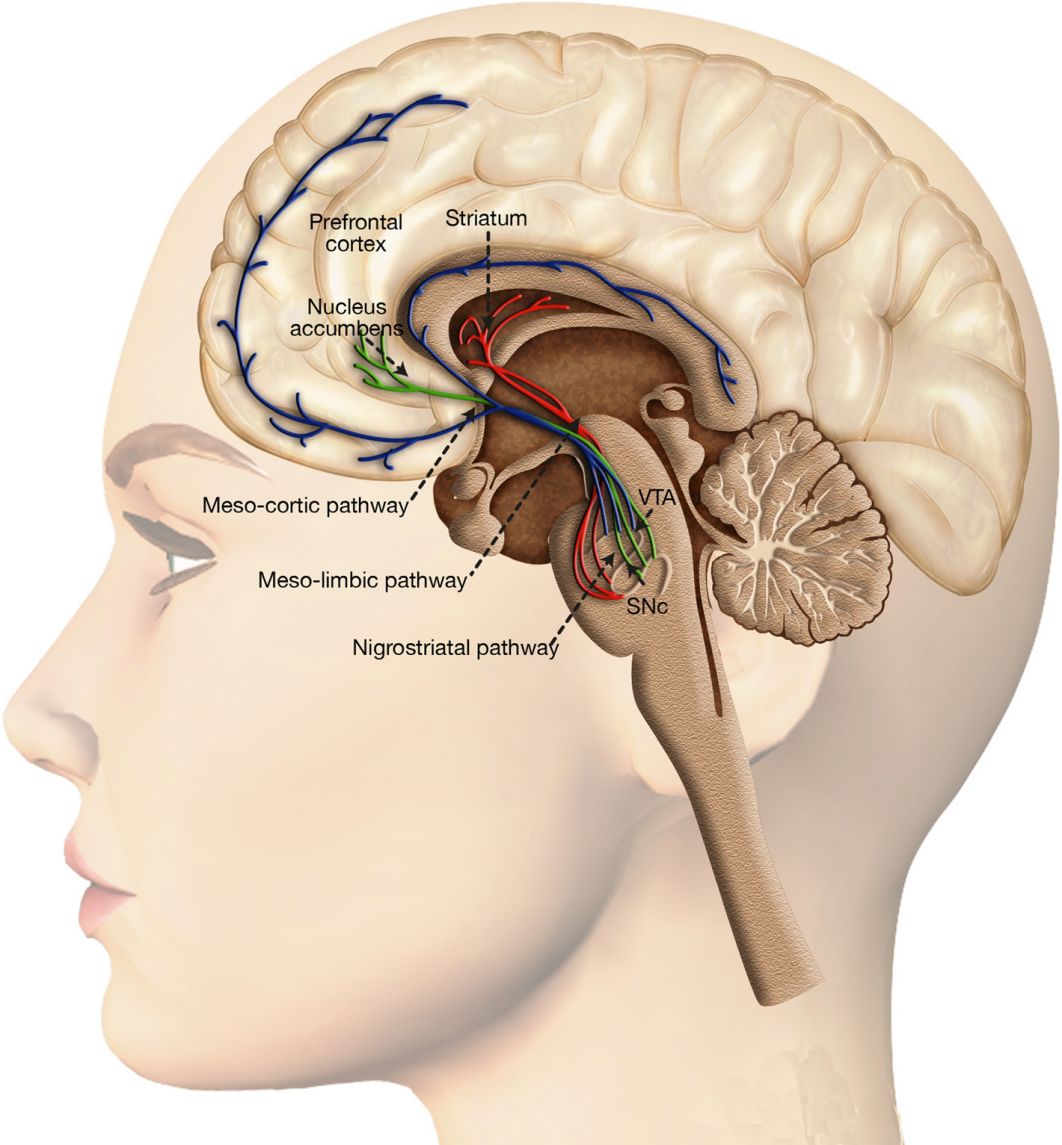
**Dopaminergic system and reward processing.** Dopaminergic neurons are located in the midbrain structures substantia nigra (SNc) and the ventral tegmental area (VTA). Their axons project to the striatum (caudate nucleus, putamen and ventral striatum including nucleus accumbens), the dorsal and ventral prefrontal cortex. The mesolimbic dopamine pathway mediates the psychopharmacology of reward, whether that is a natural high or a drug-induced high, and is sometimes referred to as the pleasure center of the brain, with dopamine as the pleasure neurotransmitter.

Another hypothesis is that compulsion is due to neuroadaptations in the mesocorticolimbic dopamine system and glutamatergic corticolimbic circuitry in which the dopamine projections are embedded (Figure 1) [9]. This was inspired from studies on the role of cellular events underlying *Synaptic Plasticity processes* of learning and the behavioral effects of drugs [10]. By Synaptic plasticity we mean alterations at the level of the synapse, typically measured using electrophysiological methods (e.g. changes in AMPA/NMDA ratio). In drug addiction neural circuits are exposed to changes imparted/transmitted by the addicted drugs, leading to the craving characteristic of addiction [11]. Evidence for these changes can be seen in several forms of plasticity in brain regions known to affect motivation, and reward processing [12-14]. These adaptations range from altered neurotransmitter levels to altered cell morphology and changes in transcriptional activity [15]. Morphologically, most of these neuroadaptations have been found in the mesocorticolimbic system and the extended amygdala [13,15,16]. Since these regions plays prominent roles in regulation of mood and processing of natural rewards, plasticity has been strongly linked to addictive behavior [7].

### Addiction and plasticity

In the field of drug addiction, several theories have been used to explain the relation between plasticity and addiction. According to the previously mentioned incentive sensitization theory, repeated drug exposure sensitizes the incentive-motivational properties of drugs and drug-related cues. These changes cause the sensitized nucleus accumbens (NAc) to release dopamine (DA) following exposure to drug or related cues (Figure 1). This would manifest behaviorally as excessive craving for the drug. This can be modeled experimentally by measuring drug-seeking behaviors in response to cues paired with drug administration in animals [17]. It is worth noticing that sensitization is universal for reward both drug and non-drug induced [18].

Another theory that can link plasticity to addiction is the opponent process theory [19]. In short, it hypothesizes that there are two processes that occur during repeated experiences: 1. affective or hedonic habituation, and 2. affective or hedonic withdrawal [20]. This theory uniquely suits the pattern of opiate abuse where the early euphoric effects represent the affective habituation process, while in case of abstinence the withdrawal manifestations drive the addict to seek drug intake [21].

What appears to be an expansion of opponent process theory is the allostatic model of brain motivational systems [19]. In Allostasis we have two opposing processes, a within-system adaptation and a between-system adaptation. In the within-system process, the drug elicits an opposing, neutralizing reaction within the same system in which the drug elicits its primary and unconditioned reinforcing actions, while in the between-system process, different neurobiological systems that the one initially activated by the drug are recruited. Recently, George et al., showed interest in alterations in the dopaminergic and corticotropin releasing factor systems as within-system and between-system neuroadaptations respectively, that underlie the opponent process to drugs of abuse [22]. They hypothesized that repeated compromised activity in the dopaminergic system and sustained activation of the CRF–CRF1R system with withdrawal episodes may lead to an allostatic load contributing significantly to the transition to drug addiction. Acute withdrawal from drugs of abuse produces opponent process-like changes in reward neurotransmitters in specific elements of reward circuitry associated with the mesolimbic dopaminergic system and recruitment of the extended amygdala and CRF stress systems that motivationally oppose the acute hedonic effects of drugs of abuse. Such changes in the dopamine and CRF these brain systems associated with the development of motivational aspects of withdrawal are hypothesized to be a major source of neuroadaptive changes that drive and maintain addiction. Decreased dopaminergic function in the nucleus accumbens and extended amygdala may participate in the habituation of the a-process, i.e., or the acute reinforcing efficacy of natural rewards and drugs of abuse, whereas recruitment of the CRF–CRF1 system and possibly dynorphin/κ opioid system in the CeA, BNST, and VTA during withdrawal may participate in the emergence of the b-process, i.e., or negative emotional state that drives the motivation to seek drugs. Although some evidence suggests that the dopaminergic and CRF systems may closely interact with each other, research in this domain is scarce. It is unknown whether the initial activation of the dopaminergic system in the VTA (a-process) is required for the increase in CRF release in the extended amygdala and VTA (b-process) in drug-dependent and withdrawn subjects that leads to compulsive drug seeking and increased craving for the drug. As such, repeated withdrawal episodes and sustained activation of the CRF-CRF1R system may lead to an allostatic load contributing significantly to the transition to drug addiction.

A third theory to describe the role of neuroplasticity in addiction is the recruitment of habit-based neurocircuitry throughout repeated drug exposure [14]. In cocaine self administration in animals, there are changes in glucose metabolism and levels of dopamine D2 receptor and dopamine transporter that initially affect the ventral striatum, these changes later expand to affect the dorsal striatum [23]. This progression of plasticity from ventral to dorsal striatum may account for the transition from goal- to habit-based learning in older works [24].

An alternative hypothesis stating that neural areas supporting electrical self stimulation of the brain (ESSB), constitute a basic emotional brain circuit, this is what we can call the SEEKING/EXPECTANCY system. This system changes the individual’s attitude towards the environment, and creates expectancy states that paves the road for future rewards [25]. What seems interesting about this hypothesis is that the activation of SEEKING is experienced by organisms as rewarding per se, leading to ESSB without the need for any traditional form of consummatory activity and explicit sensory rewards [25,26]. Based on drug administration, microinjections and lesion studies, the ML-DA system seems to constitute an essential component of the rewarding effects of MFB stimulation [27]. Even in the case of opioids (which have separate rewarding effects), animals tend to self-administer compounds that increase DA levels in ML areas [25,26,28]. Although intracranial self-administration studies revealed the role of many other neurochemicals different from DA in brain reward and approach functions [28], the ML-DA system remains the principal neurochemical that seems to be involved in the whole trajectory of the SEEKING system. Recently, Alcaro and Panksepp proposed that addicts are generally characterized by an abnormal expression of SEEKING [29]. If depression is characterized by a general reduction of SEEKING urges, addiction may be described as a re-organization of such a weakened emotional disposition around specific and often dangerous environmentally driven activities. In their affective neuroethogical view, addiction is the result of an “emotional shrinkage”, due to an ever increasing recruitment of the SEEKING emotional disposition by memories of addictive rewards and desires to alleviate the dysphoria arising from drug withdrawal [30].

Several lines of evidence support the conclusion that the brain’s mesencephalic dopamine system is involved in sensing and responding to rewards of a wide range of modalities. However, the precise role of dopamine in reward processing is still a matter of investigation [26,31,32]. Initially it was believed that dopamine carries a pleasure or hedonic signal, indicating the reward value of experienced objects [32,33]. This explanation has proven to be too simplistic. The receipt of rewards can evoke increased dopaminergic activity, but numerous conditions exist for which this does not hold. Several hypotheses have been proposed to replace the hedonia hypothesis [27,33]. This review focuses on the theory that activity changes in dopamine neurons encode an error in the prediction of the time and amount of immediate and future rewards (the prediction error hypothesis). Increased dopaminergic activity is hypothesized to indicate that the immediate or future prospect for reward was better than previously expected, while decreased dopaminergic activity signals the converse [34]. This signal may be used to learn to predict rewards as well as to guide decisions aimed at acquiring rewards [27,35].

### Dopaminergic system and reward processing

In the adult brain, dopaminergic (DA) neurons are an anatomically and functionally heterogeneous group of cells, localized in the mesencephalon, diencephalon and the olfactory bulb [32,36]. However, nearly all DA cells reside in the ventral part of the mesencephalon (Figure 1). Mesodiencephalic DA neurons form a specific neuronal group that includes the substantia nigra pars compacta (SNc), the ventral tegmental area (VTA) and the retrorubral field (RRF). Probably, the best known is the nigrostriatal system, which originates in the SNc and extends its fibers into the caudate-putamen nucleus and plays an essential role in the control of voluntary movement [37,38]. More medial to this pathway are the mesolimbic and mesocortical DA system, which arise from DA neurons present in the VTA and are involved in emotion-related behavior including motivation and reward [33,39,40]. The mesolimbic DA system include the DA cells of the VTA that project mainly to the nucleus accumbens, to the olfactory tubercle but they also innervate the septum, amygdala and hippocampus. In the mesocortical DA system, the VTA extends its fibers in the prefrontal, cingulate and perirhinal cortex. Because of the overlap between these two systems they are often collectively referred to as the mesocorticolimbic system (Figure 1) [41,42].

In humans, there are relatively few neurons in the SN and VTA, numbering less than 400,000 in the SN and roughly 5,000 in the VTA [36,43]. While the number of neurons is small, the projections from individual neurons are quite extensive and hence have profound effects on brain function. A typical midbrain DA neuron is thought to have total axonal length (including collaterals) totaling roughly 74 cm [36]. Synaptic connections are equally extensive, with 500,000 terminals common for an individual neuron [36]. In the striatum, where DA terminals are at their densest, they account for approximately 20% of all synapses in the structure [44,45].

From their different nuclei, DA axons progress medially where they join and project through the median forebrain bundle (MFB) to the internal capsule [36]. From the internal capsule, the axons branch off to form synapses in their target locations [36]. Substantia nigra neurons terminate principally in the caudate and putamen nuclei (striatum), forming the nigrostriatal system. DA axons originating in the VTA terminate largely in the ventral part of the striatum; a region called the nucleus accumbens (NAc), and is the principal components of the mesolimbic system [36].

The diverse physiological actions of DA are mediated by at least five distinct G protein-coupled receptor subtypes [46,47]. Two D1-like receptor subtypes (D1A-1D and D5) couple to the G protein Gs and activate adenylyl cyclase [46,47]. The other receptor subtypes belong to the D2-like subfamily (D2, D3, and D4) and are prototypic of G protein-coupled receptor that inhibit adenylyl cyclase and activated K + channels [46,47].

The DA receptors have a similar pattern to the distribution of projection neurons [32,48]. The relative concentration of D1-like receptors compared to D2 receptor is higher in the prefrontal cortex, whereas the concentration of D2-like receptors is elevated in the caudate nucleus, putamen, and nucleus accumbens of humans [46,49]. Although D1 and D2 receptors have opposite effects at the molecular level, they often act synergistically when more complex outputs are taken into account [50,51].

DA acts via G-protein-coupled receptors in a typical neuromodulatory fashion [52]. DA release sites are placed immediately outside the synaptic cleft [53,54]. Once released, DA diffuses in the extracellular fluid from which it is slowly cleared as a result of reuptake and metabolism [55]. DA does not directly affect the conductance of receptive membranes but modifies their response to afferent input [56,57]. These three aspects (extrasynaptic release, G-protein-coupled receptor signal transduction and a modulatory mechanism) contribute to a basic feature of DA transmission, that is, the long delay occurring between stimulus-bound activity (burst firing) and functional changes in the receptive elements. It has been estimated that, following electrical stimulation of DA neurons, a change in activity is recorded in striatal neurons after a delay of approximately 300 ms [58]. Although burst firing of DA neurons occurs in response to motivationally relevant stimuli [59], it is unlikely that these phasic DA signals influence, to any significant extent, the behavioral response (mediated by fast transmitting pathways) to the same stimulus that triggered them [60,61]. Thus, a more realistic view of the role of DA in responding involves DA as a delayed amplifier of responding, affecting the behavioral impact of stimuli that follow the one that triggered its release [60,61].

### Self-administered drugs affect dopaminergic system

A separate line of study identifying DA systems in reward processing began with an investigation into the reinforcing properties of drugs of abuse. Most findings support the conclusion that addictive drugs share the common property of enhancing the effect of midbrain DA function, particularly at the level of their terminals in the nucleus accumbens [62,63]. Cocaine is a monoamine uptake blocker which binds with greatest affinity to dopamine transporters. DA uptake transporters, in turn, are the dominant mechanism for removal of dopamine from synapses. Blockade of the transporters, therefore, greatly enhances DA’s efficacy. It is this effect that is believed to be the cause of cocaine addiction [64]. Amphetamines work via a similar method. In addition to blocking DA uptake transporters, amphetamines are also taken up by the transporters, and through intracellular effects induce a reversal of transporter function [65,66].

The result is a net release of DA by uptake transporters, and hence increased DA function. Other drugs of abuse have more indirect effects on DA function [67,68]. Alcohol is believed to affect brain function primarily by enhancing the function of GABA receptors, the primary inhibitory receptors in the brain [69]. Ethanol is known to reduce the firing rate of neurons in the substantia nigra pars reticulata [70], which in turn are believed to limit the firing of DA neurons [70,71]. By inhibiting these neurons, alcohol causes a net increase in DA cell firing, and increased DA release in the striatum and nucleus accumbens [72,73]. Opiates cause a similar release of DA in the striatum [74], both through disinhibition in the VTA and through direct effects on DA terminals [74,75]. Furthermore, blocking opioid receptors in either the VTA or nucleus accumbens reduces heroin self-administration [76]. Self-administration of nicotine is also blocked by infusion of dopamine receptor antagonists or by lesion of dopamine neurons in the nucleus accumbens [77]. Thus the DA system has been proposed to be critically involved in nicotine addiction as well [78]. The proposal that the DA system may be part of a final common pathway for the reinforcing effects of drugs of abuse is very appealing and fits in nicely with the literature on brain self-stimulation [79]. Furthermore, chronic exposure to drugs of abuse causes longterm adaptations in cAMP concentrations, tyrosine hydroxylase production, DA expression, receptor coupling to G proteins, and basal firing rate of VTA-DA neurons [80,81]. These mechanisms have been thought to underlie addiction and contribute to relapse to drug taking following periods of abstinence [17,82,83].

**Figure 2:**
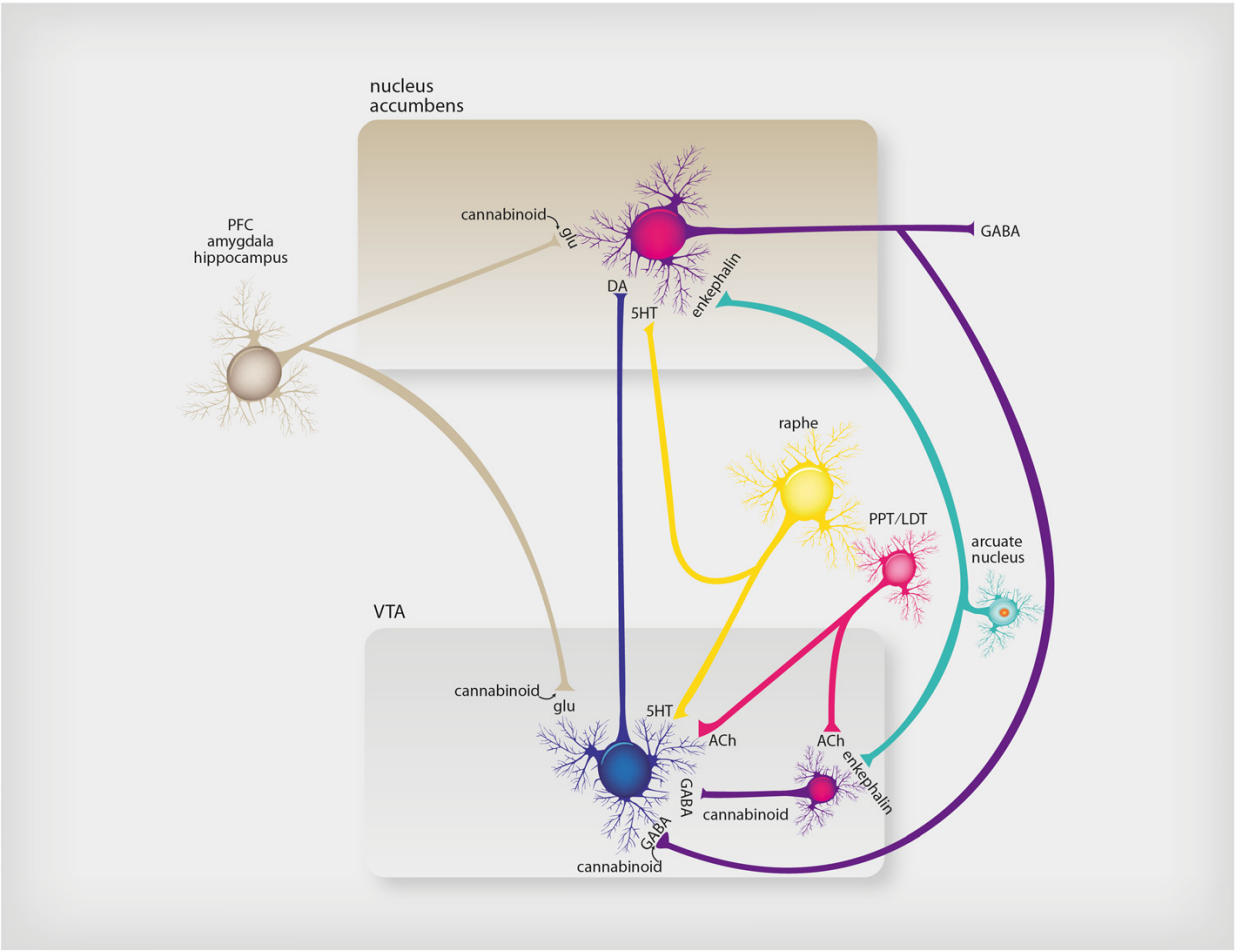
**Neurotransmitter regulation of reward-seeking behavior.** The common pathway of reward-seeking behavior in the brain is the mesolimbic dopamine pathway. This pathway is modulated by many naturally occurring substances in the brain in order to deliver normal reinforcement to adaptive behaviors (such as eating, drinking, sex) and thus to produce “natural highs,” such as feelings of joy or accomplishment. These neurotransmitter inputs to the reward system include the brain’s own morphine/heroin (i.e., endorphins such as enkephalin), the brain’s own cannabis/marijuana (i.e., anandamide), the brain’s own nicotine (i.e., acetylcholine), and the brain’s own cocaine/amphetamine (i.e., dopamine itself), among others. The numerous psychotropic drugs of abuse that occur in nature bypass the brain’s own neurotransmitters and directly stimulate the brain’s receptors in the reward system, causing dopamine release and a consequent “artificial high.” Thus alcohol, opiates, stimulants, marijuana, benzodiazepines, sedative hypnotics, hallucinogens, and nicotine all affect this mesolimbic dopaminergic system.

Drug addiction is not as simple a phenomenon as the link to the DA system would suggest, however. Mice bred without DA transporters, which are the substrate for cocaine effects on the DA system, are still capable of developing cocaine addiction [84,85]. This discovery suggested that cocaine’s effects on serotonergic and noradrenanergic transporters may also serve an important role in drug abuse [86]. This idea is further supported by the fact that enhanced serotonergic function reduces alcohol self-administration [87,88]. Regardless, while the exact mechanisms of drug abuse and drug addiction are unclear, dopamine has been found to play a critical role in both phenomena, thereby strengthening the connection between brain dopamine systems and reward processing (Figure 2).

It seems that the traditional neural ‘reward’ system can be expanded to include two separate, but interconnecting systems, the limbic system in the incentive sensitization of drugs, and the prefrontal cortex (PFC) in regulating inhibitory control over drug use. Preliminary studies provide consistent evidence of a relationship between prolonged drug administration, neuroadaptations of the PFC (specifically the three PFC-striatothalamic circuits, the DLPFC, OFC and ACC), and the persistence of drug-seeking behaviors. Neuroimaging studies revealed that cocaine induced behavioral deficits are associated with structural abnormalities in the OFC and ACC, and hypoactivity of frontal cortical regions, specifically the ACC and PFC. Opiate addiction imparts diminished ability on decision-making. In this situation, neuroimaging studies showed abnormal neural responses in the PFC; they revealed attenuated activity in the ACC, with altered responses within the DLPFC and OFC. The dysfunction in these frontal regions was found to be associated with deficits in executive function and decision making ability in opiate-dependent individuals. The defective decision making would –undoubtedly- endanger the addict life who will make improper decisions in different situations. Alcohol dependence was associated with reduced levels of cognitive inhibitory control, impulsive behaviors and risk-taking decision-making skills. Neuroimaging studies of alcohol-dependent individuals revealed reduced DLPFC brain volume matter, which was supported by functional neuroimaging studies, which found that changes in impulse control are coupled by hypoactivity of the DLPFC. So, it seems that alcohol-dependent individuals carry the risk of having defective brain circuitry involved in the ability to prevent risky situations. This in turn would minimize the possibility of remaining abstinent and may help explain the high rates of relapse amongst alcohol-dependent individuals [89].

A large body of literature indicates that the shell subregion of the NAc has an important role in processing the primary motivating properties of rewarding and aversive stimuli [90]. Psychostimulants preferentially induce dopamine release in the shell [91], and animals will self-administer dopamine agonists directly to into this region [92]. Pharmacologic inhibition of the shell increases motivated behavior and hedonic responses to taste stimuli [93]. Consistent with these findings, Wheeler and colleagues, 2011 observed – through fast-scan cyclic voltammetry to examine real-time dopamine release in rats experiencing a sweet taste cue that predicted delayed cocaine availability and during self-administration- that dopamine release in this region, but not the core subregion, is rapidly elevated by palatable, and reduced by unpalatable, taste stimuli [94]. Furthermore, they showed that these rapid fluctuations in release can be altered by devaluation from learned associations, specifically the predictive and temporal relationship of the taste cue to cocaine availability. However, a rapid dopamine release was observed during cocaine self-administration and for cues for immediate cocaine delivery (either tastants or audiovisual).

### Dynorphin system and dopamine

The dynorphin-like peptides seems to be integrated in the brain reward system. Previous studies indicate that stimulation of kappa-opioid receptors leads to a negative emotional state by inhibiting the release of dopamine in the striatum. Kappa-Opioid receptor antagonists have potent antidepressant-like effects [95], moreover, it has been suggested that chronic drug intake induces neuroadaptations in the brain dynorphin system that inhibit drug induced dopamine release. Although, the increased production of dynorphin-like peptides may initially counteract the effects of drugs of abuse, these same adaptations would have negative effects when drug intake ceases leaving the track for the unopposed neuroadaptations imparted by the dynorphins. It is worthy to notice that kappa-opioid receptor agonists may attenuate drug withdrawal symptomatology by decreasing glutamatergic, GABAergic, or noradrenergic transmission in the brain [96]. As can be seen drug intake would induce adaptations in the dynorphin system mostly in the caudate putamen, globus pallidus, and the ventral pallidum [97]. Recent works revealed that these areas play a pivotal role in regulating mood states besides their known role for controlling motor functions. These data would introduce dynorphins as important palyers in reward system and inturn, investigating their role would be helpful in elucidating further therapies for drug abuse.

### Individual variations

During the last years, genetic diversity in human population has been a crucial topic in clinical research [98]. It has been hypothesized that common genetic variants may contribute to genetic risk for some diseases and that they might influence the subject’s response to drug abuse. Recently, it has been shown that inter-individual variations are evident in the field of drug rewarding [99]. In 1999, Volkow *et al*., linked the intensity of euphoria to the amount of dopamine release following D2 stimulation [100]. These findings showed variations among tested subjects. In another report, a correlation between the release of dopamine in response to amphetamine and drug seeking behavior has been demostrate [101]. An fMRI study correlated self rating of alcohol intake with striatal activity; this may show that striatal activation can affect subjective feelings and drug reward. The multiple researches showing the decrease D2 receptor availability, need further justification whether this is an effect of drug abuse, or inherent subjective character predisposing to addiction [102].

### Hypocretin/Orexin system and the reward system

Hypocretin/orexin (Hcrt) neurons are solely located in the hypothalamus, particularly in its perifornical, dorsomedial and lateral portions [103,104]. Hcrt fibers widely project throughout the brain and generally have excitatory effects on their postsynaptic cells [105-107]. Hcrt neurons regulate arousal and have been show to be implicated in food reward and drug-seeking behavior [105]. Anatomically, orexin neurons are well-positioned to alter reward functioning [103,104]. Hcrt neurons project to reward-associated brain regions, including the nucleus accumbens (NAc) and VTA, and Hcrt directly activates VTA-DA neurons through Hcrt-1 receptor [108]. This indicates a possible role for Hcrt in reward function and motivation, consistent with previous studies implicating Hcrt in feeding. In fact, the activation of Hcrt neurons was shown to be strongly linked to preferences for cues associated with drug and food rewards [109]. Dopaminergic neurons that originate in the VTA and project into the forebrain, particularly the NAc, have classically been identified as the ‘reward pathway’ [32]. Drugs of abuse stimulate this pathway. ICV or local VTA infusions of Hcrt have been shown to reinstate drug-seeking or food-seeking behavior in rodents [109,110]. Conversely, the subcutaneous morphine (μ-opioid receptor agonist)-induced place preference and hyperlocomotion observed in wild-type mice were abolished in mice that lacked the prepro-Hcrt gene [111], and injections of an Hcrt-1 receptor antagonist into the VTA block the development of morphine-conditioned place preference [111]. In vivo injection with a selective PKC inhibitor chelerythrine chloride or 2--3-1-methyl-1H-indol-3-ylmaleimide HCl (Ro-32-0432) into the ventral tegmental area (VTA) significantly suppressed the place preference and increased levels of dopamine in the nucleus accumbens (NAcc) induced by intra-VTA injection of Hcrt [112]. These results strongly support the idea that activation of the orexin-containing neuron in the VTA leads to the direct activation of mesolimbic dopamine neurons through the activation of the PLC/PKC pathway via G(q11)alpha or Gbetagamma-subunit activation, which could be associated with the development of its rewarding effect.

Recent work has provided interesting insights into the cellular and molecular mechanisms underlying these effects by showing that Hcrt-1 input to the VTA potentiates NMDAR (N-methyl-d-aspartate receptor)-mediated neurotransmission through a protein kinase C-dependent insertion of NMDARs in VTA dopamine neuron synapses in slice preparations [113,114] Furthermore, in vivo administration of an Hcrt-1 receptor antagonist blocks locomotor sensitization to cocaine and occludes cocaine-induced potentiation of excitatory currents in VTA dopamine neurons [113,114]. These results suggest an important role for Hcrt signalling in the VTA in the neural plasticity associated with reward, and indicate that Hcrt also contribute to cocaine-induced psychomotor sensitization and reward-seeking. These findings highlight the key role of orexin in the mechanisms of reward and drug addiction. Consistently, prepro-Hcrt-knockout mice are less susceptible than wild-type animals to developing morphine dependence, as measured by physical withdrawal responses [115]. Interestingly, some narcolepsy patients with daytime sleepiness who were treated with amphetamine-like stimulants and/or sodium oxybate (γ-hydroxybutyrate, also known as GHB) for a long time rarely developed drug abuse [116]. These observations indicate the strong functional interaction between Hcrt pathways and the DA system [117].

In rat studies, following extinction training cocaine-seeking was reinstated via re-exposure to drug related cues. However, this cue-induced reinstatement of cocaine-seeking or context-induced reinstatement of cocaine-seeking [118] was blocked by systemic administration of 20 or 30 mg/kg SB (ORX-1 blocker) [119]. Similar results, however, were not obtained on using OxR2 antagonist 4pyridyl methyl (S)-tert-leucyl 6,7-dimethoxy-1,2,3,4-tetrahydrosisquinoline (4PT), indicating a unique role of orexin signaling specifically at OxR1 in cocaine-seeking [119]. Moreover, SB has been shown to significantly reduce self-administration of ethanol, nicotine, high-fat food, and sucrose [120], as well as ethanol intake in alcohol-preferring outbred rats [121]. As can be seen Orexin system plays an important role in rewarding.

### Orexin and abstinence

The function of orexin system seems to be related to their site. So, reward-seeking functions are associated primarily with orexin cells in LH, whereas arousal- and stress-related processes are linked with orexin neurons in the DMH and PeF [122]. Several studies support this opinion. For example, PeF and DMH orexin neurons show increased Fos activation during waking compared to sleep [123]. On the other hand, neuroleptics preferentially activate LH orexin neurons [124]; chronic ethanol consumption increased the area of orexin mRNA expression in LH but not DMH/PeF. These differential functions of orexin neurons indicate different networking related to arousal or reward. So, LH orexin cells project to VTA or medial prefrontal cortex (mPFC) [124]. While, PeF/DMH orexin neurons are innervated by other hypothalamic regions [117].

### Corticotropin-releasing factor (CRF) and orexin/hypocretin

Recently, it has been suggested that the N/OFQ (nociceptin/orphanin FQ) and Orx/Hct neuropeptide systems interact with CRF system. N/ OFQ inhibit the activity of Orx/Hcrt neurons [125]. This effect will lead to the hypothesis that N/OFQ also modulates Orx/Hcrt functions, including behavioral response to stress, anxiety, reward, and addiction. Investigation of these interactions will be an important focus of future research on stress-regulatory neuropeptidergic systems [126].

### Histaminergic system and reward

Although dopaminergic system represent the cornerstone in rewarding, other neurotransmitter systems such as endogenous opioids, glutamate, GABA, acetylcholine, serotonin, adenosine, endocannabinoids, orexins, galanin and histamine have been found to modulate the rewarding and psychomotor effects of addictive drugs [127]. Several studies revealed that the histaminergic system modulates mesolimbic dopamine transmission. Moreover, it seems to modify the rewarding properties of drugs. Supporting this hypothesis is the finding that dopamine anatagonists failed to prove clinical efficiency in treating drug abuse. This has been confirmed by the finding that the H inverse agonist BF2.649 (Tiprolisant) enhanced histamine neuronal activity and decreased methamphetamine-induced locomotor activity [128].

### The brain histaminergic system

The tuberomamillary nucleus (TM) consists of relatively few neurons, which forms the main source of histamine in the brain. However, histaminergic neurons have a wide network of projections that can reach most brain areas. But there is inter-regions variablitiy regarding density of these projections with the highest density in the hypothalamic nuclei. H receptors are G protein-coupled receptors (GPCRs): the. Three of the four H receptors 1–3 are widely distributed in the mammalian central nervous system. H receptors are mainly located postsynaptically and mediate excitatory actions on whole-brain activity. H1 receptor is coupled to G q/11 leading to activation of phospholipase C, with the two second messengers, DAG and and IP(3). H2, on the other hand, are coupled to Gs and activate adenylyl cyclase, PKA and cAMP-response element binding protein (CREB). On the contrary, H3 receptors are coupled to G i/o with inhibit adenylyl cyclase. This makes them inhibitory receptors. They can inhibit the synthesis and release of various neurotransmitters including DA, noradrenalin, GABA and acetylcholine [129].

### Linking the histaminergic to dopaminergic systems

High densities of H2 and H3 receptors are found striatum (including the NAc) in mice, rats, monkeys and humans [130]. Moreover, striatal cholinergic interneurons contain H1 [131]. Despite the great controversy, several reports found that antagonizing H1 can induce addiction-like effects in animals and humans through enhancing the release of dopamine. However, the relation between the two systems is not that simple as histamine can act on different neuronal systems to either inhibit or activate midbrain dopamine activity. Through H1 receptors possibly located on striatal cholinergic interneurons, histamine can activate the mesolimbic system. Inversely, histamine can decrease dopamine transmission through H 3 receptors located either presynaptically on dopamine terminals or postsynaptically on GABAergic neurons in the striatum [132].

### Central Ghrelin system and reward

Ghrelin system has important link to the control of food intake and energy balance [133]. Ghrelin system includes those pathways affected by stimulation of ghrelin receptor, GHS-R1A (growth hormone secretagogue receptor 1A). GHS-R1A is wide spread in brain; including the hypothalamus, brainstem, tegmentum and hippocampus. The “central ghrelin signalling system” is the term best describe pharmacology of this receptor, since it shows activity in the absence of ghrelin ligand [134]. The first notion of GHS-R1A was in the 1980s when a peptide called growth hormone-releasing peptide 6 (GHRP6), which was discovered to be a stimulant of the hypothalamo-pituitary growth axis [135]. Later on, their ligand GHS-R1A, was described by Merck & Co. Group. The discovery, that the hypothalamic cells activated by GHRP-6 was another milestone in this system discovery. The precise mechanisms of ghrelin affecting rewards remains need further research. However, it seems to be related to cholinergic–dopaminergic reward system. GHS-R1A is expressed pre and post-synaptically in the VTA [136] as well as on cholinergic neurons in the LDTg [137]. Dickson et al. [137] suggested that the central ghrelin signalling system act as enhancer of reward reinforcers through altering the set point of the dopaminergic neurons in the VTA. More interesting is the finding that GHSR1A shows activity in absence of ligand. This would question whether it is ghrelin itself that provides signal to enhance the reward mechanism. Indeed GHS-R1A was found to be regulated independently of ghrelin via heterodimerization to the dopamine D1-like receptor [138]. It remains not yet known how the dopamine D1 receptor influences central ghrelin signalling and the physiological relevance of this dimerization remains to be determined. Moreover, ghrelin system has been linked to the rewarding of alcohol [139,140], cocaine, amphetamine [141], and palatable/rewarding food [142]. Collectively these studies imply that central ghrelin signalling, including the GHS-R1A may constitute a novel target for development of treatment strategies for addictive behaviours [139].

### Galanin and reward system

The gut peptide galanin was discovered in the 80s [143]. This discovery was followed by others denoting that galaninis are also distributed throughout brain. These ligands proved to be linked to multiple critical functions including feeding behavior, pain modulation, seizure, learning and memory [144]. There are three galanin receptors: GalR1, GalR2 and GalR3 [145]. They are G protein-coupled and can activate Gi and Go proteins [146]. Besides activating Gi and Go proteins as GalR1-3, GalR2 also activates Gq proteins [146] and can increase calcium signaling and the activity of downstream effectors such as PKC [147]. This would denote complex functions of different galanin receptor subtypes.

### Galanins and dopamine system

Galanin decreases stimulation-evoked dopamine release in rat striatal slices through a mechanism that involves Gi proteins [148]. This is consistent with the ability of galanin to decrease glutamate, but not GABA release in striatal slices. Moreover, intraventricular administration of galanin can increase DOPA accumulation in the striatum, NAc and olfactory tubercles and reduce locomotor activity in rats [149]. Since the net effect on behavior is hypoactivity, the authors suggest that the increase in DOPA accumulation results from a decreased release of dopamine, relieving an autoreceptor mediated tonic inhibition of dopamine synthesis. The effect of galanin on DOPA accumulation also occurs when galanin is microinjected into the VTA, but not the NAc, suggesting that the VTA is a primary site of action for the effects of galanin on the mesolimbic system [149]. Consistent with this hypothesis, galanin decreases locomotor activity in rats when injected either into the ventricle, the VTA or the hypothalamus [150]. Taken together, these results suggest that galanin effects in the VTA can decrease the activity of the mesolimbic system.

Although galanin has no effect on the number of TH immunoreactive neurons on its own, treatment with dibutyryl cAMP increases the number of TH-positive neurons, and this effect is decreased by galanin. These cultures express GalR1, GalR2, and, to a lesser extent, GalR3 receptor mRNA, but treatment with dibutyryl cAMP specifically increases GalR1mRNA levels. Therefore, galanin may inhibit midbrain dopamine activity through a reduction of TH activity mediated through activation of GalR1 receptors. While GalR1 knockout mice and wild type mice do not differ in baseline locomotion [151].

### Galanin modulates addiction-related behaviors

In accord with the ability of galanin to modulate midbrain dopamine activity, a number of studies have shown that the galanin system modulates drug-related behaviors. For example, administration of galanin into the lateral ventricles attenuates the development of conditioned place preference for morphine in mice [152]. Consistent with this finding, knockout mice lacking the galanin peptide, unlike congenic wild type mice, are sensitive to the locomotor stimulant properties of morphine and show increased morphine conditioned place preference [153]. Several other links between the galanin system and opioid addiction have been reported. Chronic, systemic injection of morphine in rats down regulates galanin expression in the extended amygdala in a mu-opioid receptor-dependent manner [154], whereas GalR mRNA is increased in the LC during opiate withdrawal [155]. Moreover, single nucleotide polymorphisms in the human galanin gene are associated with heroin addiction [156]. Galanin has also been shown to modulate the behavioral response to psychostimulants. Mice lacking the galanin peptide are more sensitive to the rewarding effects of cocaine as measured by conditioned place preference [157]. Consistent with this effect, transgenic mice that over express galanin are less sensitive to the stimulant effects of amphetamine, compared to wild type mice [158]. Taken together, these data suggest that the overall effect of galanin in the brain is to decrease behavioral responses to morphine and psychostimulants.

In contrast to morphine and psychostimulants, galanin can increase alcohol consumption under several experimental conditions. Administration of galanin either into the third ventricle or into the PVN of the hypothalamus enhances voluntary alcohol intake in normal rats, an effect also observed in the presence of food and in rats selected for high alcohol intake [159]. The opposite effects of galanin on morphine, amphetamine and cocaine locomotion and reward compared to alcohol intake suggest that different brain areas mediate these two sets of responses. It is tempting to speculate that galanin effects on hypothalamic circuits involved in feeding are important for its effects on alcohol intake, whereas modulation of systems converging on the mesolimbic dopamine systemmay be critical for its effects on psychotimulant-and opiate-related behaviors. The ability of galanin to alter norepinephrine, serotonin, acetylcholine and glutamate release may indirectly alter the activity of dopamine neurons, leading to modulation of drug-related behaviors. Taken together, a large, convergent body of evidence suggests that endogenous galanin exerts a tonic inhibition on multiple neurotransmitter systems that may mediate drug self-administration and withdrawal symptoms. Future studies focusing on the ability of galanin to modulate the mesolimbic pathway in vivo and in vitro will be necessary to gain a better understanding of how pharmacological agents targeting the galanin system might be used to treat drug addiction in human subjects [160].

## Conclusions

The past decade has brought an enormous wealth of knowledge on human reward processing using functional brain imaging. Much progress has been made in understanding the neural substrates of human reward processes, but much remains to be learned, and much integration needs to go on among information at the molecular, cellular, systems, and behavioral levels (Figures 1 and 2).

The pursuit of mechanisms underlying reward has been hampered by the limitations of current animal models and thus requires that basic investigators exchange ideas with those involved in human experimental biology and clinical research. It is clear that neurotransmitters other than DA must play important roles in regulating hedonic states and even in reward-related learning (Figure 1).

Consumption of rewards (e.g., palatable food, mating, cocaine) produces hedonic consequences which initiate learning processes that consolidate liking the rewarding goal. Motivational states such as hunger, sexual arousal, and perhaps early symptoms of drug withdrawal increase the incentive salience of reward-related cues and the reward itself. The greater the hunger, the greater the likelihood that behavioral sequences aimed at obtaining food will be initiated and carried to conclusion despite distractions and obstacles that may arise. Positive reinforcement involves an increase over time in the frequency of behaviors that lead to a reward. Understanding the neurobiology of the addictive process allows a theoretical psychopharmacological approach for treating addictive disorders, one that takes into account biological interventions aimed at particular stages of the illness (Figure 2).

## Competing interests

None of the authors have actual or potential conflict of interest including any financial, personal or other relationships with other people or organizations that could inappropriately influence, or be perceived to influence, our work.

## Authors’ contributions

OAC, XCS, SSL and EMR designed, conducted the literature review and drafted most of the manuscript. MS, SM, AEN and MMG performed the literature review and the drafting of the manuscript. All authors read and approved the final manuscript.
